# The impact of regional environmental governance efficiency on residents’ life satisfaction—an empirical analysis of panel data from 21 cities in Guangdong Province, China (2001–2023)

**DOI:** 10.3389/fpubh.2025.1697459

**Published:** 2026-01-16

**Authors:** Hui Jin, Shijian Wu, Shujian Zhang

**Affiliations:** 1 Faculty of Humanities and Social Sciences, Macao Polytechnic University, Macao, China; 2 Faculty of Business, The Hong Kong Polytechnic University, Hong Kong, China; 3 School of Government, Shenzhen University, Shenzhen, China

**Keywords:** China, environmental governance efficiency, Guangdong, mental health, public administration, residents’ life satisfaction

## Abstract

**Background:**

The study of the mental health of residents in the prosperous southern regions of China, which have experienced long-term rapid growth, is a very meaningful topic. Studies that approach the issue from the perspective of the relationship between environmental governance efficiency and residents’ consumption level are quite rare.

**Methods:**

This study explores the nexus of environmental governance efficiency and residents’ life satisfaction in Guangdong Province, China, through a psychological and public administration lens, using panel data from 21 cities (2001–2023). Life satisfaction, a critical indicator of mental health, is proxied by the ratio of consumption expenditure to disposable income, reflecting residents’ economic behavior and wellbeing. Environmental governance efficiency, measured via the super-efficiency SBM model of Data Envelopment Analysis (DEA), captures the psychological benefits of effective public administration by improving living conditions through sustainable environmental policies. Two-way fixed effects models, validated by the Hausman test were employed.

**Results:**

We find a significant positive relationship between governance efficiency and life satisfaction (FE1, FE2, and FE4, *p* < 0.001; FE3, *p* < 0.01), underscoring the role of high-quality governance in fostering mental wellbeing. Environmental governance efficiency, health insurance coverage, per capita GDP, fiscal self-sufficiency, and economic openness significantly enhance residents’ life satisfaction in Guangdong by increasing consumption expenditure, though industrial production and government scale show negative effects, and regional disparities persist. The fixed-effect model, validated by the Hausman test, confirms these findings, highlighting the importance of high-quality governance and social services in supporting mental wellbeing.

**Conclusion:**

In Guangdong, a region marked by rapid urbanization and economic dynamism, efficient environmental governance mitigates pollution and enhances urban livability, positively impacting residents’ mental health. This study demonstrates that efficient environmental governance in Guangdong Province significantly enhances residents’ life satisfaction by improving living conditions and supporting mental wellbeing, offering a replicable model for sustainable development in rapidly growing economies.

## 1 Introduction

In contemporary Chinese society, particularly in economically advanced regions like Guangdong Province, the material living standards of residents have reached a level of relative security. Decades of rapid economic growth, driven by China’s reform and opening-up policies, have significantly enhanced public infrastructure, urban amenities, and access to daily conveniences. These developments have laid a robust foundation for improving the quality of life. However, as material needs are increasingly met, public attention has shifted toward the pursuit of high-quality social governance to further enhance residents’ sense of wellbeing and mental health. This transition reflects a broader societal evolution, where the expectations of Chinese citizens have moved beyond mere economic prosperity to encompass modernized, efficient, and responsive governance systems. The growing emphasis on governance quality underscores its critical role in fostering societal progress and ensuring sustainable development in China’s rapidly urbanizing and market-oriented regions.

The concept of life satisfaction, as a multidimensional measure of wellbeing, has gained prominence in academic and policy discussions globally. In China, where economic growth has been a cornerstone of national development, life satisfaction is increasingly tied to both material and non-material factors (1). Among these, consumption levels serve as a key indicator of residents’ economic behavior and overall wellbeing (2, 3). In recent years, insufficient domestic consumption demand has emerged as a notable challenge in China, potentially exerting a negative influence on the collective mental health of its population (4). This issue is particularly pronounced in Guangdong Province, a coastal economic powerhouse characterized by a robust market economy and a large, diverse population. As one of China’s most dynamic regions, Guangdong offers a unique case study for examining how consumption patterns reflect and influence residents’ life satisfaction (5, 6). To capture this relationship empirically, this study employs the ratio of consumption expenditure to disposable income as the dependent variable, providing a quantifiable measure of life satisfaction that aligns with residents’ economic realities.

Central to the discourse on life satisfaction is the role of regional governance, particularly in the provision of public goods and services. China’s governance model, often described as “big government, small society,” places significant responsibility on state institutions to deliver public goods that underpin social development and residents’ wellbeing (7, 8). Within this framework, environmental governance has emerged as a critical dimension of public administration, especially in regions like Guangdong, where rapid industrialization and urbanization have heightened public expectations for sustainable and effective environmental policies (9, 10), environmental governance efficiency not only reflects a government’s administrative capacity but also directly impacts residents’ physical and mental health through the quality of their living environment. In this study, we focus on the efficiency of environmental governance across 21 cities in Guangdong Province as the independent variable to assess the level of public governance. Rather than solely measuring governance by budgetary allocations, we adopt a comprehensive approach that accounts for both desired and undesired outputs of environmental efforts. By calculating an efficiency value that incorporates these outputs, we aim to provide a nuanced representation of local governance performance and its impact on residents’ life satisfaction.

Guangdong Province serves as an ideal setting for this study due to its status as one of China’s most market-driven and reform-oriented regions (11, 12). The province has been at the forefront of China’s economic transformation, benefiting from early market liberalization and global integration. Its residents, accustomed to a relatively high standard of living, have correspondingly high expectations for governance, particularly in areas such as environmental management, which directly affect their quality of life (13). The efficiency of environmental governance in Guangdong is not merely a matter of administrative competence; it is a critical determinant of residents’ health, wellbeing, and overall satisfaction with their local government. For instance, effective environmental policies can mitigate pollution, improve air and water quality, and enhance urban livability, all of which contribute to residents’ mental and physical health (14, 15). Conversely, inefficiencies in governance, such as misallocated resources or failure to address environmental challenges, can undermine public trust and diminish life satisfaction (16, 17). By focusing on environmental governance efficiency, this study seeks to uncover how well local governments in Guangdong translate resources into tangible outcomes that enhance residents’ wellbeing.

The significance of this study extends beyond Guangdong Province. For decades, the Chinese government has looked to Guangdong as a model for regional development, replicating its successful strategies in other parts of the country. The province’s experience in balancing economic growth with social and environmental considerations offers valuable lessons for China’s broader modernization agenda. As concerns about residents’ life satisfaction and mental health gain traction across the country, understanding the factors that influence these outcomes in Guangdong could inform national policy frameworks. The findings of this study, based on an empirical analysis of panel data from 21 cities in Guangdong over the period from 2001 to 2023, have the potential to yield policy recommendations with far-reaching implications. By identifying the mechanisms through which environmental governance efficiency shapes life satisfaction, this research aims to contribute to the broader discourse on high-quality development and modernized governance in China. Moreover, the study’s focus on a quantifiable measure of governance efficiency provides a robust methodological framework for evaluating public administration in other regions, both within China and in other rapidly developing economies (18).

This paper investigates the interplay between regional environmental governance efficiency and residents’ life satisfaction in Guangdong Province, using the ratio of consumption expenditure to disposable income as a proxy for wellbeing. By analyzing panel data from 21 cities over a 23-year period, we aim to elucidate how effective environmental governance contributes to residents’ quality of life and mental health. The findings are expected to offer actionable insights for policymakers seeking to enhance social governance and promote sustainable development in China’s most economically vibrant regions.

This study adopts a behavioral economics perspective, viewing residents’ consumption behavior as an outward expression of psychological wellbeing. In high-income societies, individuals with stronger mental wellbeing tend to exhibit greater confidence in their economic future and are more inclined toward consumption rather than precautionary saving (5, 19). Environmental quality has repeatedly been shown to have causal effects on mental health and subjective wellbeing in China: air pollution significantly increases depressive symptoms and psychological distress. Efficient environmental governance reduces exposure to pollutants, which numerous studies have causally linked to increased depression, anxiety, and psychological distress in China (20, 21). By lowering these environmental stressors, high-quality governance decreases precautionary saving motives triggered by perceived health risks, thereby increasing the consumption-to-income ratio. This behavioral shift directly reflects improved subjective wellbeing and preventive mental health in a public health sense, rather than clinical diagnoses. Thus, the ratio of consumption expenditure to disposable income reflects both material satisfaction and psychological stability, offering an observable behavioral proxy for residents’ life satisfaction and mental health.

## 2 Literature review

The academic community has conducted extensive foundational research on the relationship between economic development and individuals’ life satisfaction and happiness. These studies have established a robust interdisciplinary research foundation for further investigations into human mental health (22–28). Interdisciplinary approaches integrating economics, psychology, and sociology have highlighted the role of relative income, social capital, and cultural context in shaping subjective wellbeing (29–31), these findings underscore the complexity of the relationship between economic prosperity and mental health, paving the way for nuanced investigations into how economic policies can be designed to optimize both individual and societal wellbeing (32–35).

Numerous studies have explored the complex interplay between mental health, socioeconomic factors, and environmental conditions, particularly during periods of economic hardship. Economic downturns, such as cyclical crises and recessions, disproportionately impact the middle class and blue-collar workers, who often face job insecurity, reduced income, and financial strain. These challenges can lead to a measurable decline in overall life satisfaction, contributing to increased stress, anxiety, and other mental health issues across affected populations (36–39).

In densely populated urban areas, housing conditions play a critical role in shaping individuals’ wellbeing (40–45). Inadequate, overcrowded, or unaffordable housing can exacerbate feelings of instability and dissatisfaction, further undermining mental health. For many in the middle class, employment status is a key determinant of financial security, directly influencing income levels and consumption patterns (46–48), stable employment fosters a sense of economic confidence and supports access to essential resources, such as healthcare, leisure, and education, all of which contribute to greater life satisfaction. Conversely, unemployment or underemployment can trigger a cascade of negative effects, including diminished self-esteem, financial stress, and reduced quality of life, all of which are closely linked to deteriorating mental health (38, 49–51).

Moreover, the ripple effects of economic and environmental stressors extend beyond the individual, impacting families and communities (52) for instance, financial pressures may strain interpersonal relationships, while substandard living conditions in urban settings can limit access to safe, restorative spaces, further compounding mental health challenges (53, 54) Addressing these interconnected issues requires a holistic approach, including policies that promote economic stability, equitable housing, and accessible mental health resources to support vulnerable populations (55, 56).

Existing research demonstrates that environmental quality positively affects life satisfaction and mental health, particularly through reduced exposure to pollutants (57, 58). In China, studies show air pollution correlates with lower subjective wellbeing and higher depression rates (59). Governance aspects, such as policy implementation, amplify these effects (60). However, few examine governance efficiency via output-oriented models like DEA, especially in panel data from dynamic regions like Guangdong, where our study fills a gap by quantifying efficiency’s role in consumption-mediated wellbeing. Recent research increasingly highlights the connection between environmental governance and residents’ subjective wellbeing. Studies in China have shown that improvements in air and water quality, green space, and pollution control directly enhance residents’ happiness and perceived life quality (61, 62). Moreover, effective governance that ensures equitable access to environmental public goods fosters trust in local authorities, which in turn supports mental wellbeing (63, 64). However, most existing studies rely on cross-sectional or subjective survey data, while few adopt efficiency-based approaches to measure governance performance. By applying the super-efficiency SBM model, this study advances the literature by linking objective measures of governance efficiency to behavioral indicators of life satisfaction over two decades of panel data.

Recent years’ research highlights the complex interplay between consumption expenditure levels and mental health outcomes, revealing both positive and negative associations depending on the nature and context of spending (65–68). Some study notes that financial strain from high consumption expenditure increases perceived stress, mediating negative mental health outcomes like anxiety and depression (69, 70), materialistic consumption, characterized by prioritizing expenditure on material goods, consistently correlates with poorer mental health, including higher rates of depression and anxiety (71). Conversely, spending on experiential purchases, such as travel or events, is linked to improved wellbeing and life satisfaction (72). Studies also indicate that relative consumption, where individuals compare their spending to others, exacerbates psychological distress, particularly in contexts of income inequality or financial strain (4, 73). Furthermore, compulsive spending, such as on digital platforms or subscriptions, is associated with increased stress and depressive symptoms (74–77). Sustainable consumption aligned with personal values, however, appears to enhance mental health by reducing anxiety and fostering a sense of purpose (78, 79). These findings underscore the nuanced role of consumption expenditure in shaping mental health, influenced by social comparison, financial pressures, and the type of expenditure (80–82).

## 3 Variables, data, and method

### 3.1 Environmental governance efficiency as the explanatory variable

Our paper examines the relationship between local government governance levels and residents’ life satisfaction in China’s wealthiest coastal provinces (83, 84) Environmental governance is one of the toughest tasks for local governments and one of the most closely tied to residents’ lives. We chose environmental governance efficiency (*Efficiency*) as the explanatory variable because it goes beyond just financial investment in environmental protection or green awareness. Instead, it uses the DEA model to estimate governance efficiency, incorporating both desired and undesired outputs. This variable offers a more objective reflection of local governments’ true governance capabilities. We estimated local environmental governance efficiency score with super-efficiency SBM method. The efficiency of the effective DMU in traditional non-super-efficiency DEA model cannot be further distinguished. Super-efficiency model by removing the evaluated DMU from the reference set, that is, the efficiency of the evaluated DMU is obtained by referring to the frontier formed by other DMU, solves the problem that the efficiency of effective DMU cannot be distinguished. Suppose the total number of decision units (DMU) in period T is K, and each DMU uses M input factors and produces I desired outputs and R undesired outputs, $x_{k}\in R^{M}$ , $\;y_{k}\in R^{I}$ and $b_{k}\in R^{R}$ respectively represent the input vector, expected output vector and unexpected output vector of the k DMU, then, the input–output of the k DMU in period t is expressed as $\left(x_{k}^{t},y_{k}^{t},b_{k}^{t}\right)$ . Define the production possibility set constructed by other DMU other than $\mathrm{DM}U_{k}$ as Equation (1):


$$P^{t}=\{\begin{array}{l} (x^{t},y^{t},b^{t})|x^{t}\geq \sum_{j=1,j\neq k}^{K}x_{j}^{t}\lambda_{j}|,y^{t}\\ \leq \sum_{j=1,j\neq k}^{K}y_{j}^{t}\lambda_{j}|\;b^{t}\geq \sum_{j=1,j\neq k}^{K}b_{j}^{t}\lambda_{j}|\lambda_{j}\geq 0 \end{array}\}$$ (1)

Where, $\lambda_{j}$ is the weight coefficient vector (intensity vector), here we assume that scale returns are variable (i.e., VRS), so the sum of weight coefficients of all decision making units is equal to 1, i.e., $\sum_{j=1,j\neq k}^{K}\lambda_{j}=1$ . Here, DMU is each district in Guangdong Province, and the input variable of each area is environmental input. The expected output variable is waste water utilization rate and solid waste treatment rate, and the unexpected output variable is sulfur dioxide and nitrogen oxide. The environmental input variable represents the annual fiscal expenditure of each city government on environmental management and pollution control, as recorded in the Guangdong Statistical Yearbook. The expected output variables include (1) the wastewater utilization rate, defined as the ratio of wastewater treated by treatment plants to the total wastewater effluent during the reporting period, and (2) the solid waste treatment rate, defined as the proportion of general industrial solid waste comprehensively utilized relative to the total amount generated plus the previously stored volume. The undesired outputs are sulfur dioxide and nitrogen oxide emissions. Therefore, *M* = 1, *I* = 2, *R* = 2.

The super-efficiency SBM efficiency value of decision unit K k∈{1,2,⋯,K} can be obtained by solving the following programming problem:


$$\begin{array}{l} IE_{\text{SuperSBM}}^{t}(x_{k}^{t},y_{k}^{t},b_{k}^{t},\lambda )=\\ \min\frac{1+(1/M)\sum_{m=1}^{M}(s_{m}^{x,-}/x_{m,k}^{t})}{1-[1/(I+R)][\sum_{i=1}^{I}(s_{i}^{y,+}/y_{i,k}^{t})+\sum_{r=1}^{R}(s_{r}^{b,-}/b_{r,k}^{t})]} \end{array}$$ (2)

$$\begin{array}{l} \;s.t.\;\sum_{j=1,j\neq k}^{K}x_{m,j}^{t}\lambda_{j}-s_{m}^{x,-}\leq x_{m,k}^{t}\\ \;\sum_{j=1,j\neq k}^{K}y_{j}^{t}\lambda_{j}+s_{i}^{y,+}\geq y_{i,k}^{t}\\ \;\sum_{j=1,j\neq k}^{K}b_{j}^{t}\lambda_{j}-s_{r,k}^{b,-}\leq b_{r,k}^{t}\\ \;s^{x,-}\geq 0,s^{y,+}\geq 0,s^{b,-}\geq 0,\lambda \geq 0,\sum_{j=1,j\neq k}^{K}\lambda_{j}=1\\ \;m=1,2,\cdots ,M;\;i=1,2,\cdots ,I;\;r=1,2,\cdots ,R \end{array}$$

Among them, $IE_{\text{SuperSBM}}$ stands for regional efficiency. $s_{m}^{x,-}$ , $s_{i}^{y,+}$ , $s_{r}^{b,-}$ respectively represent the relaxation variables corresponding to input variables, expected output variables and non-expected output variables. To solve Equation 2, we use the method of Charnes and Cooper (92) to convert the equation into the linear programming problem.


$$\begin{array}{l} IE_{\text{SuperSBM}\_L}^{t}(x_{k}^{t},y_{k}^{t},b_{k}^{t},\lambda )=\min\tau \\ +(1/M)\sum_{m=1}^{M}(S_{m}^{x,-}/x_{m,k}^{t}) \end{array}$$ (3)

$$\begin{array}{l} s.t.\;1=\tau -[1/(I+R)]\\ \left[\sum_{i=1}^{I}(S_{i}^{y,+}/y_{i,k}^{t})+\sum_{r=1}^{R}(S_{r}^{b,-}/b_{r,k}^{t})\right]\\ \;\sum_{j=1,j\neq k}^{K}x_{m,j}^{t}\Lambda_{j}-S_{m}^{x,-}\leq \tau x_{m,k}^{t}\\ \;\sum_{j=1,j\neq k}^{K}y_{j}^{t}\Lambda_{j}+S_{i}^{y,+}\geq \tau y_{i,k}^{t}\\ \;\sum_{j=1,j\neq k}^{K}b_{j}^{t}\Lambda_{j}-S_{r,k}^{b,-}\leq \tau b_{r,k}^{t}\\ \;S^{x,-}\geq 0,S^{y,+}\geq 0,S^{b,-}\geq 0,\Lambda \\ \geq 0,\tau >0,\sum_{j=1,j\neq k}^{K}\Lambda_{j}=\tau \\ \;m=1,2,\cdots ,M;\;i=1,2,\cdots ,I;\;r=1,2,\cdots ,R \end{array}$$

Then the optimal solution of the original nonlinear programming problem (2) can be got by solving the optimal solution of equation 3 of linear programming.

Accordingly, we can also get the efficiency of each input–output variable.


$$DE_{k,t}^{\text{in}}=(x_{k,t}^{\text{in}}-s_{k,t}^{\text{in}})/x_{k,t}^{\text{in}},,DE_{k,t}^{\mathrm{uo}}=(b_{k,t}^{\mathrm{uo}}-s_{k,t}^{\mathrm{uo}})/b_{k,t}^{\mathrm{uo}}DE_{k,t}^{\mathrm{do}}=y_{k,t}^{\mathrm{do}}/(y_{k,t}^{\mathrm{do}}+s_{k,t}^{\mathrm{do}}).$$

**Figure 1:**
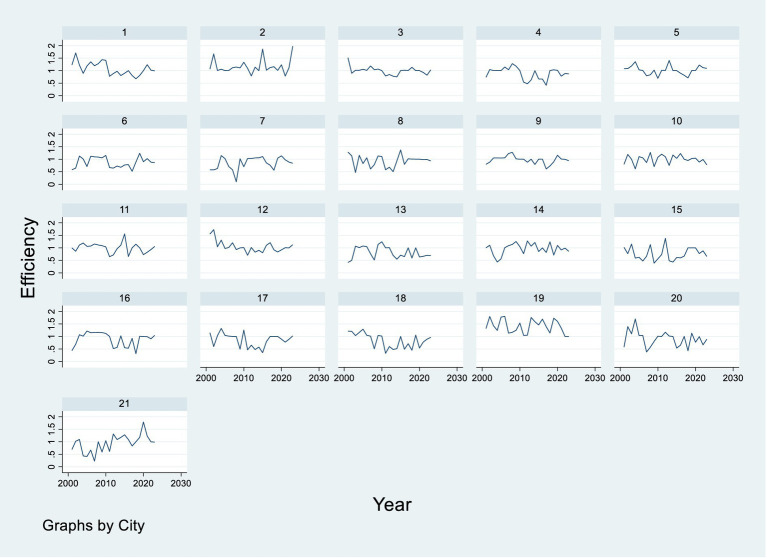
Environmental governance efficiency score by city in Guangdong Province, China between 2001 and 2023.

Among them, $DE_{k,t}^{\text{in}}$ , $DE_{k,t}^{\mathrm{uo}}$ , $DE_{k,t}^{\mathrm{do}}$ respectively represents the efficiency of input variable, expected output variable and unexpected output variable. The larger the value is, the higher the efficiency of the input or output factor is Figure 1 shows the time trends of environmental governance efficiency values in different regions in Guangdong province from 2001 to 2023.

### 3.2 The ratio of consumption expenditure to disposable income as the dependent variable

**Figure 2:**
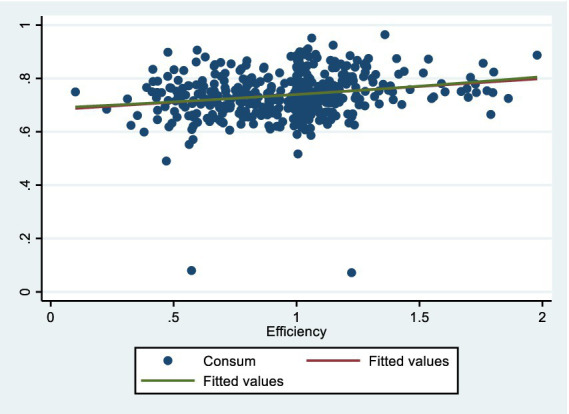
Scatter plots and fitting curves of the relationship between the explanatory variable and the explained variable.

**Figure 3:**
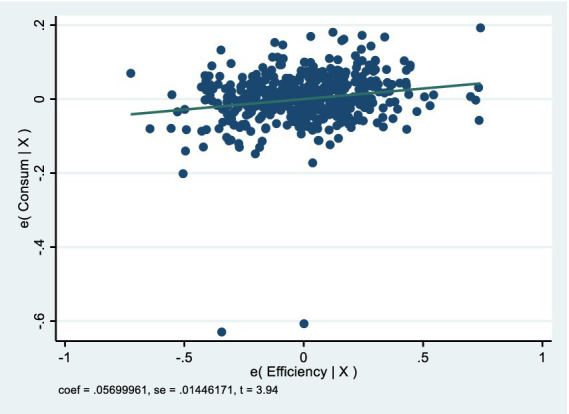
Partial correlation graphs between the explanatory variable and the explained variable.

**Figure 4:**
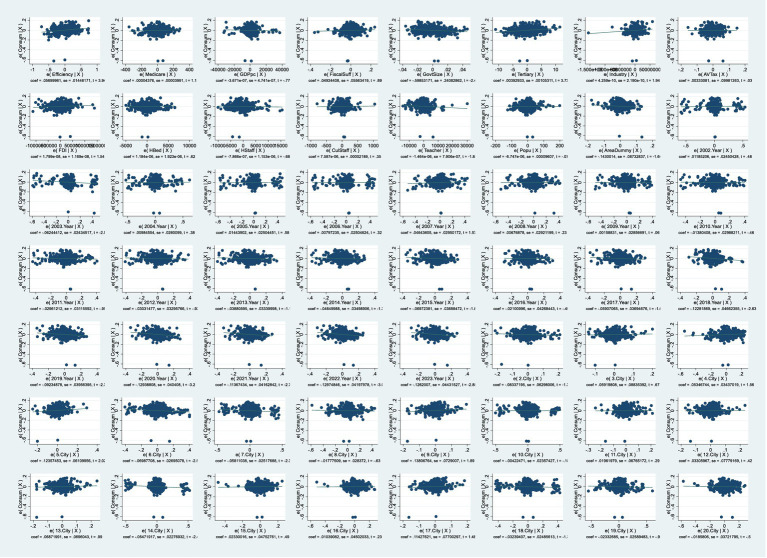
Partial correlation graphs between all variables and the explained variable.

We focus on gaging residents’ life satisfaction through domestic consumption levels. Consumption depends on people’s income, but per capita disposable income is not a spot-on measure of their true feelings, it is more of a decent macroeconomic figure. What really matters is how much of that disposable income people actually spend, as that is the foundation for ordinary folks’ genuine sense of happiness in life. So, the ratio of consumption spending to disposable income is a solid economic indicator of regional residents’ life satisfaction, serving as the material basis for mental wellbeing in economically advanced areas. Although survey-based subjective wellbeing (SWB) measures (85) are common, they suffer from cultural response biases, recall errors, and limited availability in long-term (23-year) Chinese panel datasets at the city level. In contrast, the consumption-to-income ratio is an objective, revealed-preference indicator that is particularly appropriate in high-growth East Asian contexts where high precautionary saving is driven by uncertainty (86). Recent studies similarly use objective indicators to predict subjective wellbeing in China because they avoid the subjectivity of surveys while capturing real behavioral outcomes (87, 88). We explicitly follow this emerging objective approach. That is why we picked this ratio (*Consum*) as the dependent variable—it helps level out differences in spending caused by varying baseline incomes across regions. Figure 2 shows the basic scatter plots and fitting curves of the relationship between the explanatory variable and the explained variable. Figures 3, 4 respectively show the partial correlation graphs between the explanatory variables and each control variable and the explained variable.

### 3.3 Control variables

Since China’s social security system is still developing, ordinary people face hefty medical expenses. A big chunk of a family’s savings often ends up going toward healthcare costs. So, when measuring residents’ life satisfaction, we need to consider whether they have solid medical coverage. Without health insurance, people tend to cut back on daily spending, which drags down their overall happiness (89, 90). That is why, in this study, our top control variable is residents’ health insurance status. We treat the proportion of people with medical coverage (*Medicare*) as the most critical control variable. We use per capita GDP (*GDPpc*), total industrial output (*Industry*), and population size (*Popu*) as the most common control variables for economic development levels. Beyond the economic scale, the structure of economic development has a major impact on residents’ employment and quality of life in a region. We’ve included indicators like the proportion of the service sector (*Tertiary*), value-added tax ratio (*AVTax*), and economic openness (*FDI*) as control variables. China operates as a government-led society, and the size of the government plays a big role in both economic development and residents’ lives. Government size can be measured in two ways: positively, through a region’s fiscal self-sufficiency (*FiscalSuff*), and negatively, through the proportion of government administrative expenses for daily operations (*GovtSize*). Additionally, public services directly affect residents’ life satisfaction, with education and healthcare being the two most influential factors, aside from environmental governance. We’ve chosen metrics like the number of hospital beds (*HBed*), healthcare workers per 10,000 people (*HStaff*), cultural workers (*CulStaff*), and primary and secondary school teachers (*Teacher*) as control variables to gage local government public service provision. In Guangdong Province, there’s a stark contrast in socioeconomic development between regions (*AreaDummy*), so we typically divide the Pearl River Delta and other parts of the province into two distinct areas to account for regional differences. Table 1 reports the statistical descriptions of each variable.

**Table 1:** Statistical summarization table for variables.

Variable	Obs	Mean	SD	Min	Max
Consum	483	0.7381057	0.083831	0.0718079	0.9640311
Efficiency	483	0.9624187	0.2776283	0.100572	1.978275
Medicare	483	269.2921	248.1954	2.5865	1396.109
GDPpc	483	45293.07	36818.41	4178.964	195230.2
FiscalSuff	483	0.5512865	0.2510316	0.1354	1.168413
GovtSize	483	0.1203371	0.0319032	0.0509	0.2204
Tertiary	483	42.41123	8.53727	24.44	73.33784
Industry	483	4.56e+07	7.27e+07	334,569	4.92e+08
AVTax	483	0.2223428	0.0732873	0.0505267	0.4450526
FDI	483	272287.6	889112.3	1,680	7,300,994
HBed	483	17682.25	16594.9	2,231	117,071
HStaff	483	31769.74	34068.3	6,377	247,839
CulStaff	483	734.265	639.5841	215	4,113
Teacher	483	41759.73	22294.63	8,116	151,365
Popu	483	514.9735	338.8426	128.45	1882.7
AreaDummy	483	0.4285714	0.4953847	0	1

### 3.4 Data

All variables in the regression model come from publicly available, objective data. The data spans from 2001 to 2023, which is the most recent publicly available data for Guangdong Province that allows for a strongly balanced panel dataset. Annual data is sourced from the Guangdong Statistical Yearbook and the statistical yearbooks of various cities in Guangdong Province. Data used to estimate environmental governance efficiency, specifically regarding urban waste management (solid, liquid, and gas), comes from the China Urban Statistical Yearbook, while other environmental governance data is drawn from the annual environmental governance reports of cities in Guangdong Province.

### 3.5 Regressions

This study conducts an empirical analysis of the panel data of 21 cities in Guangdong Province over 23 years. Due to the large differences among different regions in the province, there may be omitted variables that do not change over time, so we adopted the Two-way fixed effects model:


$$\begin{array}{l} \text{lnConsu}m_{\mathrm{it}}=\beta_{0}+\beta_{1}\ln\text{Efficienc}y_{\mathrm{it}}+\beta_{2}\text{Medicar}e_{\mathrm{it}}\\ +\text{δControl}s_{\mathrm{it}}+\mu_{i}+\gamma_{t}+\varepsilon_{\mathrm{it}}\;(i=1,\cdots ,21;t=1,\cdots 23) \end{array}$$

For the selection of the model and the test of its robustness, we adopt the Hausman test.

## 4 Results

In Table 2, we find that environmental governance efficiency (Efficiency) has a significant positive impact on the proportion of consumption expenditure (Consum). Environmental governance efficiency is a true reflection of local governance quality, while the ratio of consumption expenditure to disposable income serves as a key economic indicator of residents’ mental wellbeing in a region. This shows that in Guangdong, a province with a highly developed market economy, high-quality public governance services provided by the government can effectively boost residents’ life satisfaction, thereby strengthening the foundation for their mental health. This also represents a fundamental expectation of people in modern society for the modernization of national governance.

**Table 2:** Regressions result.

Variables	(1)	(2)	(3)	(4)	(5)
	OSL	FE1	FE2	FE3	FE4
Efficiency	0.915*** (0.349)	1.371*** (0.521)	1.164*** (0.443)	0.823** (0.391)	
L-Efficiency					0.796*** (0.304)
Medicare	0.271	0.603*	1.149*	0.543	0.788**
	(3.199)	(0.365)	(0.688)	(1.982)	(0.394)
GDPpc	0.119**	0.137***	0.274**	0.185*	0.203**
	(0.059)	(0.051)	(0.131)	(0.111)	(0.101)
FiscalSuff	1.472*	1.191*	0.749*	0.446	0.837**
	(0.876)	(0.722)	(0.451)	(3.135)	(0.421)
GovtSize	0.817	−0.704**	−1.003		0.656
	(1.095)	(0.335)	(1.711)		(0.596)
Tertiary	−0.414	0.549*		0.213*	0.672*
	(0.112)	(0.331)		(0.127)	(0.382)
Industry	1.439	−1.079*			−0.883
	(1.304)	(0.65)			(0.679)
AVTax	2.071	1.664	2.193	−0.627	2.458
	(1.146)	(0.997)	(1.483)	(1.68)	(2.731)
FDI	0.685*	1.637***		0.942**	2.157**
	(0.411)	(0.606)		(0.428)	(0.995)
HBed	1.108	0.713	0.882*		1.349*
	(1.557)	(2.014)	(0.531)		(0.787)
HStaff	0.304*	1.018*		0.944	0.516
	(0.183)	(0.609)		(1.157)	(1.86)
CulStaff	2.113	3.044	3.791		2.425*
	(0.995)	(1.561)	(2.828)		(1.402)
Teacher	1.337***	2.714**		1.605*	1.928*
	(0.495)	(1.292)		(0.961)	(1.102)
Popu	−4.192	3.708	−3.113		2.943
	(5.779)	(3.451)	(4.68)		(3.567)
AreaDummy	2.256	1.944**		2.076*	1.381*
	(2.474)	(0.883)		(1.258)	(0.755)
Constant	2.927**	1.778*	2.703	1.814**	2.019**
	(1.394)	(1.071)	(2.499)	(0.825)	(0.874)
R-sq	0.4319	0.2771	0.7076	0.6771	0.5893

*p < 0.05, **p < 0.01, ***p < 0.001.

Paying for resident health insurance (Medicare), used as the main control variable, positively affects consumption expenditure. Both fixed-effect regression results show a significant impact, suggesting that people with health insurance coverage tend to spend more, leading to greater life satisfaction. Per capita GDP (GDPpc) has a significant positive effect in every regression model, which aligns with general economic development patterns. Fiscal self-sufficiency (FiscalSuff), an effective measure of a local government’s financial health, shows a significant positive impact in the regression results. Meanwhile, the scale of government (GovtSize), as a negative fiscal indicator, though not consistently significant, shows a negative impact in fixed-effect regressions, as expected. The level of service sector development (Tertiary) has a weakly significant positive effect, while the total industrial production (Industry), which heavily impacts the environment, shows a certain degree of negative effect. The value-added tax ratio (AVTax), an important indicator of manufacturing levels, is significant for Guangdong’s residents’ lives, but unfortunately, the study found no impact. We hope future research will make further adjustments. However, another key indicator for Guangdong’s open market economy—economic openness (FDI)—shows a significant positive effect in the regression results, indicating that an open economy positively influences residents’ lives. The supply of healthcare workers (HStaff), hospital beds (HBed), and primary and secondary school teachers (Teacher) all show significant positive effects in the regression results, but the number of cultural department workers (CulStaff) shows no impact. Finally, regional dummy variable (AreaDummy) reveals significant differences in development across Guangdong province.

To determine whether to use the fixed-effect model, we conducted a Hausman test on the egression results. The test result (Table 3) shows that the *p-*value is 0.0000, which strongly rejected the null hypothesis that $H_{0}:\mu_{i}$ and $x_{\mathrm{it},\;}z_{i}$ is not correlated. It means that the fixed effect model should be adopted instead of the random effect model. While the two-way fixed effects model controls for time-invariant city-specific factors and year effects, potential endogeneity—such as reverse causality where higher resident satisfaction drives better governance—cannot be fully ruled out. However, in Guangdong’s governance context, environmental policies are predominantly top-down (initiated by provincial or national directives), reducing the likelihood of resident-driven reversals (91). To further address potential reverse causality, we re-estimated the main models using the 1-year lagged environmental governance efficiency variable (L-Efficiency). The coefficient remains positive and highly significant, confirming that past governance efficiency predicts current life satisfaction rather than the reverse (see Table 3).

**Table 3:** Hausman test result.

Variable	Coefficients			
	(b) FE	(B) RE	(b-B) Difference	sqrt[diag(V_b-V_B)] Std. err.
Efficiency	1.217904	0.8913713	1.309812	0.4182586
Medicare	0.7044237	0.306489	0.6137865	0.5367354
GDPpc	0.1594642	0.1077853	0.1493618	0.0813567
FiscalSuff	1.0841276	1.503571	1.1637364	0.7162513
GovtSize	−0.6801298	0.793323	−0.6719431	0.4915244
Tertiary	0.5571933	−0.3687314	0.6374159	0.2075638
Industry	−1.103487	1.341178	−1.204045	0.5107732
AVTax	1.594732	2.142135	1.428707	0.5019178
FDI	1.728245	0.7037128	1.453206	0.6782301
HBed	0.6849251	0.9391467	0.8513841	0.4348029
HStaff	1.236684	0.5712582	1.447028	0.7726147
CulStaff	3.255418	2.443716	3.4037485	1.145904
Teacher	2.913429	1.579125	2.770183	0.6271089
Popu	3.946172	−4.327618	−3.243877	1.243018
_cons	1.9168293	2.613956	1.742812	0.7129341

b = Consistent under H0 and Ha; obtained from xtreg.

B = Inconsistent under Ha, efficient under H0; obtained from xtreg.

Test of H0: Difference in coefficients not systematic.

Chi2(5)=(b−B)’[(V_b−V_B)^(−1)](b−B)


=57.08


$$\text{Prob}>{\mathrm{chi}}^{2}=0.0000\;(V\_b-V\_B\;\text{is not positive definite}).$$

## 5 Discussing the empirical findings and their implications

From a psychological perspective, higher consumption ratios can indicate greater subjective wellbeing and reduced anxiety about future uncertainty, consistent with findings from behavioral economics that link financial confidence with emotional stability and life satisfaction. The empirical results of this study confirm a significant positive relationship between environmental governance efficiency (Efficiency) and residents’ life satisfaction, as measured by the ratio of consumption expenditure to disposable income (Consum) across 21 cities in Guangdong Province from 2001 to 2023. This finding aligns with prior research highlighting the critical role of effective public governance in enhancing subjective wellbeing. The use of the super-efficiency SBM model to estimate environmental governance efficiency provides a robust measure that accounts for both desired and undesired outputs, offering a more comprehensive assessment of governance performance than traditional budgetary metrics. The positive impact of Efficiency on Consum suggests that high-quality environmental governance, which improves air and water quality and mitigates pollution, directly enhances residents’ living conditions, thereby fostering greater life satisfaction. This is particularly relevant in Guangdong, where rapid industrialization and urbanization have heightened public expectations for sustainable environmental policies.

It is important to note that a high consumption-to-income ratio can theoretically indicate two opposite conditions: (1) higher life satisfaction and economic confidence, leading to less precautionary saving, or (2) financial stress compelling households to spend a larger portion of limited income on necessities. In Guangdong, a high-growth region with strong income growth and developed social security systems, the first interpretation is more plausible. The observed positive association between governance efficiency and consumption behavior supports the view that efficient governance enhances residents’ confidence and perceived economic security, thereby promoting psychologically healthier consumption patterns.

Contextualizing findings within Guangdong’s unique socioeconomic landscape. Guangdong’s status as a market-driven, reform-oriented region amplifies the importance of these findings. The province’s residents, accustomed to a relatively high standard of living, exhibit heightened sensitivity to governance quality, particularly in environmental management. The significant positive effect of economic openness (FDI) underscores the role of global integration in shaping residents’ wellbeing, as an open economy facilitates access to resources and opportunities that enhance consumption and life satisfaction. Conversely, the negative effect of industrial production (Industry) highlights the environmental trade-offs associated with heavy industrialization, which can degrade living conditions and offset the benefits of economic growth. These findings corroborate studies that link environmental quality to mental health outcomes, emphasizing the need for balanced development strategies that prioritize sustainability alongside economic progress.

Analyzing the role of control variables. The significant positive effect of health insurance coverage (Medicare) on consumption expenditure underscores the importance of social safety nets in fostering economic confidence and life satisfaction. Residents with access to healthcare are more likely to allocate disposable income to non-essential spending, reflecting reduced financial anxiety and improved wellbeing. Similarly, the positive effects of per capita GDP (GDPpc), fiscal self-sufficiency (FiscalSuff), and public service provisions (HStaff, HBed, Teacher) highlight the multifaceted nature of life satisfaction, where economic stability and access to essential services play complementary roles. The lack of significance for cultural workers (CulStaff) and value-added tax ratio (AVTax) suggests that these factors may have less direct influence on residents’ consumption behavior in Guangdong, though further research is needed to explore their contextual relevance.

Addressing regional disparities. The regional dummy variable (AreaDummy) reveals stark socioeconomic disparities between the Pearl River Delta and other parts of Guangdong, consistent with prior studies on regional inequality in China. These disparities underscore the need for tailored governance strategies that address the unique challenges faced by less-developed areas, where environmental governance inefficiencies may disproportionately impact residents’ wellbeing. The Two-way fixed effects model, validated by the Hausman test, effectively controls for unobserved heterogeneity, ensuring the robustness of these findings.

Limitations and future research directions. Despite its contributions, this study has limitations. The reliance on the consumption expenditure to disposable income ratio as a proxy for life satisfaction may not fully capture the multidimensional nature of wellbeing, which includes non-material factors such as social relationships and personal fulfillment. While the consumption-to-income ratio provides an objective and consistent behavioral indicator of residents’ life satisfaction, it does not fully encompass the emotional or social dimensions captured by survey-based subjective wellbeing (SWB) measures. Future studies could complement this approach by integrating survey data or psychometric indicators of mental health to triangulate behavioral and perceptual measures of wellbeing. Additionally, the study’s focus on environmental governance efficiency excludes other governance dimensions, such as education or transportation, which may also influence life satisfaction. Although the fixed effects model and Hausman test results confirm the robustness of our empirical estimates, potential endogeneity cannot be entirely ruled out. Cities with higher baseline life satisfaction may indirectly influence local governance quality. However, the use of two-way fixed effects and long-term panel data helps mitigate this concern by controlling for unobserved city-specific and temporal factors. Future research could further explore this relationship using lagged variables or instrumental variable approaches to enhance causal inference. Future research could incorporate subjective wellbeing measures, such as survey-based life satisfaction scores, to complement objective economic indicators. Moreover, extending the analysis to other Chinese provinces or developing economies could provide comparative insights into the generalizability of these findings.

## 6 Policy suggestions

Enhancing environmental governance efficiency. To enhance residents’ life satisfaction, local governments in Guangdong should prioritize improving environmental governance efficiency. This involves adopting advanced technologies and management practices to maximize desired outputs (e.g., waste water utilization and solid waste treatment) while minimizing undesired outputs (e.g., sulfur dioxide and nitrogen oxide emissions). Policymakers could invest in data-driven monitoring systems to track environmental performance and allocate resources more effectively, ensuring that governance efforts translate into tangible improvements in residents’ living environments.

Strengthening social safety nets. The positive effect of health insurance coverage on consumption expenditure highlights the need for expanded social safety nets. Policymakers should aim to increase the proportion of residents with medical coverage, particularly in less-developed areas of Guangdong, to reduce financial burdens and encourage consumption. Subsidized healthcare programs and public awareness campaigns could enhance access to insurance, fostering economic confidence and improving mental health outcomes.

Promoting sustainable economic development. Given the negative impact of industrial production on life satisfaction, local governments should promote sustainable economic development by incentivizing green industries and enforcing stricter environmental regulations. Policies that support the growth of the service sector (Tertiary) and attract foreign direct investment (FDI) can enhance economic openness while mitigating environmental degradation. For instance, tax incentives for eco-friendly businesses and international partnerships for green technology could align economic growth with environmental sustainability.

Addressing regional disparities. To address regional disparities, policymakers should implement targeted interventions in less-developed areas outside the Pearl River Delta. These could include infrastructure investments, capacity-building programs for local governments, and regional cooperation initiatives to share best practices in environmental governance. By reducing disparities in governance efficiency and economic development, policymakers can ensure more equitable improvements in life satisfaction across Guangdong.

Enhancing public service provision. The significant effects of healthcare workers (HStaff), hospital beds (HBed), and teachers (Teacher) underscore the importance of public service provision. Local governments should prioritize investments in healthcare and education infrastructure, ensuring adequate staffing and resources to meet residents’ needs. Community-based programs that promote access to healthcare and education can further enhance life satisfaction by addressing both material and non-material dimensions of wellbeing.

## 7 Conclusion

This study provides robust evidence that environmental governance efficiency significantly enhances residents’ life satisfaction in Guangdong Province, as measured by the ratio of consumption expenditure to disposable income. By analyzing panel data from 21 cities over the period from 2001 to 2023, we demonstrate that high-quality environmental governance, characterized by efficient resource utilization and reduced environmental degradation, fosters better living conditions and supports mental wellbeing. The positive effects of health insurance coverage, per capita GDP, fiscal self-sufficiency, and public service provisions further highlight the interconnected roles of economic stability, social safety nets, and governance quality in shaping residents’ quality of life.

The findings have significant implications for China’s broader modernization agenda, particularly in economically advanced regions like Guangdong. As a model for regional development, Guangdong’s experience underscores the importance of balancing economic growth with environmental sustainability and effective governance. The study’s methodological framework, leveraging the super-efficiency SBM model and Two-way fixed effects regression, offers a replicable approach for evaluating governance efficiency in other regions, both within China and in other rapidly developing economies.

To sustain and enhance residents’ life satisfaction, policymakers must prioritize environmental governance efficiency, strengthen social safety nets, promote sustainable economic development, and address regional disparities. By aligning governance strategies with residents’ expectations for a high quality of life, Guangdong can continue to serve as a blueprint for modernized governance and sustainable development in China. Future research should explore additional dimensions of governance and subjective wellbeing to further enrich our understanding of these critical issues.

## Data Availability

The original contributions presented in the study are included in the article/supplementary material, further inquiries can be directed to the corresponding author.
